# Bis{(*E*)-4-bromo-2-[(2-chloro-3-pyrid­yl)imino­meth­yl]phenolato-κ^2^ *N*,*O*}copper(II)

**DOI:** 10.1107/S1600536809027792

**Published:** 2009-07-18

**Authors:** Wen-Kui Dong, Jun-Feng Tong, Li-Li An, Jian-Chao Wu, Jian Yao

**Affiliations:** a School of Chemical and Biological Engineering, Lanzhou Jiaotong University, Lanzhou 730070, People’s Republic of China

## Abstract

In the title complex, [Cu(C_12_H_7_BrClN_2_O)_2_], the Cu^II^ center is tetra­coordinated by two phenolate O and two azomethine N atoms from two independent bidentate 4-bromo-2-[(2-chloro-3-pyrid­yl)imino­meth­yl]phenolate (*L*) ligands. In the crystal structure, the Cu^II^ atom has a distorted square-planar coordination environment. The inter­planar dihedral angles between the benzene and pyridine rings in the individual ligands are 63.83 (4) and 54.43 (3)°, indicating the pyridine ring to have considerably weaker steric hindrance.

## Related literature

For the applications of phenoxy­imines, see: John *et al.* (2007 ▶). For the structures of salen-type bis­oxime complexes, see: Dong *et al.* (2009*a* ▶,*b* ▶). Due to their chelating ability and positive redox potential, many copper(II) complexes are biologically active, see: Karmaka *et al.* (2007 ▶). For the preparation of (*E*)-[4-bromo-2-((2-chloro­pyridin-3-ylimino)meth­yl)]phenol, see: Dong *et al.* (2009*c* ▶). For bond-length data, see: Allen *et al.* (1987 ▶).
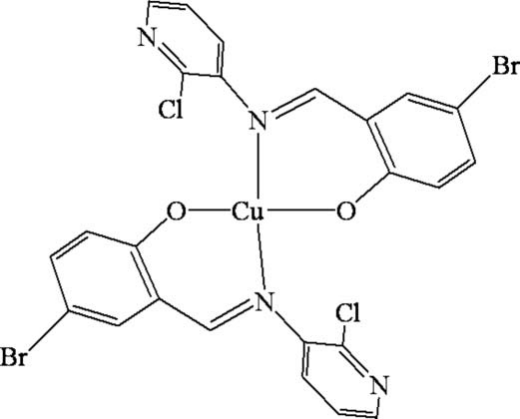


## Experimental

### 

#### Crystal data

[Cu(C_12_H_7_BrClN_2_O)_2_]
*M* *_r_* = 684.65
Monoclinic, 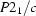

*a* = 20.406 (2) Å
*b* = 11.6378 (15) Å
*c* = 10.5787 (13) Å
β = 90.212 (2)°
*V* = 2512.2 (5) Å^3^
*Z* = 4
Mo *K*α radiation
μ = 4.29 mm^−1^
*T* = 298 K
0.43 × 0.12 × 0.05 mm

#### Data collection

Siemens SMART 1000 CCD area-detector diffractometer
Absorption correction: multi-scan (*SADABS*; Sheldrick, 1996 ▶) *T*_min_ = 0.260, *T*_max_ = 0.814
11575 measured reflections
4426 independent reflections
2340 reflections with *I* > 2σ(*I*)
*R*_int_ = 0.060

#### Refinement

*R*[*F* ^2^ > 2σ(*F* ^2^)] = 0.037
*wR*(*F* ^2^) = 0.050
*S* = 0.88
4426 reflections
316 parameters
H-atom parameters constrained
Δρ_max_ = 0.37 e Å^−3^
Δρ_min_ = −0.36 e Å^−3^

### 

Data collection: *SMART* (Siemens, 1996 ▶); cell refinement: *SAINT* (Siemens, 1996 ▶); data reduction: *SAINT*; program(s) used to solve structure: *SHELXS97* (Sheldrick, 2008 ▶); program(s) used to refine structure: *SHELXL97* (Sheldrick, 2008 ▶); molecular graphics: *SHELXTL* (Sheldrick, 2008 ▶); software used to prepare material for publication: *SHELXTL*.

## Supplementary Material

Crystal structure: contains datablocks global, I. DOI: 10.1107/S1600536809027792/hg2538sup1.cif

Structure factors: contains datablocks I. DOI: 10.1107/S1600536809027792/hg2538Isup2.hkl

Additional supplementary materials:  crystallographic information; 3D view; checkCIF report

## Figures and Tables

**Fig. 1.**
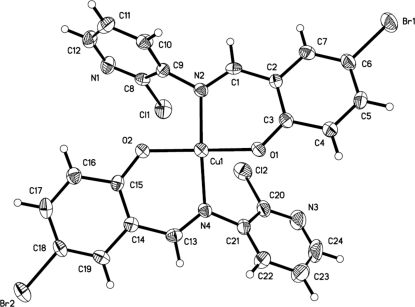
The molecular structure of the title complex with atom numbering scheme. Displacement ellipsoids for non-hydrogen atoms are drawn at the 30% probability level.

## supplementary crystallographic information

### Comment

Phenoxy-imines are a versatile class of ligands that display a truly impressive range of diverse applications spanning from bioinorganic chemistry to coordination chemistry, chemical catalysis, and materials related applications (John *et al.*, 2007). Due to their chelating ability and positive redox potential many copper(II) complexes are generally biologically active (Karmaka *et al.*, 2007). As part of our ongoing research into the complexes between transition metals and phenoxy-imine ligands, we report here the synthesis and crystal structures of the title complex, bis{(*E*)-[4-bromo-2-((2-chloropyridin-3-ylimino)methyl-κ*N*)] phenolato-κ*O*^1^}copper(II) (Fig. 1).

In the asymmetric molecule unit of the title complex, the Cu^II^ center is tetracoordinated by two phenolic O and two azomethine N atoms from two ligand (*L*^-^) units and has a distorted square-planar coordination environment, which is similar to the salen-type bisoxime complexes (Dong *et al.*, 2009*a*, Dong *et al.*, 2009*b*). It was observed that all bond lengths are within normal ranges (Allen *et al.*, 1987).

The interplane dihedral angles are found to be as follows: 63.83° between the phenyl ring (C2—C7) and pyridyl ring (N1/C8—C12), 54.43° between phenyl ring (C14—C19) and pyridyl ring (N3/C20—C24), indicating the pyridine ring having a considerable weaker steric hindrance. Besides, the dihedral angle between the coordination plane of O1—Cu1—N2 and O2—Cu1—N4 is 27.72 (3)°, indicating slight distortion toward tetrahedral geometry from the square planar structure.

### Experimental

(*E*)-[4-Bromo-2-((2-chloropyridin-3-ylimino)methyl)]phenol(HL) was prepared according to previously reported procedure (Dong *et al.*, 2009*c*). A blue solution of copper(II) acetate monohydrate (2.6 mg, 0.0013 mmol) in methanol (2 ml) was added dropwise to a pale-yellow solution of HL (8.1 mg, 0.0026 mmol) in methanol (4 ml) at room temperature. The color of the mixing solution turned to yellow immediately, then turned to brown slowly and allowed to stand at room temperature for several days. With evaporation of the solvent, dark-brown needle-like single crystals suitable for X-ray crystallographic analysis were obtained. IR: ν C=N, 1600 cm^-1^, ν Ar—O, 1242 cm^-1^, ν Cu—N, 445 cm^-1^ and ν Cu—O, 424 cm^-1^.

### Refinement

Non-H atoms were refined anisotropically. H atoms were treated as riding atoms with distances C—H = 0.93 Å (CH), and *U*_iso_(H) = 1.2 *U*_eq_(C) and 1.5 *U*_eq_(O).

### Crystal data

**Table:**

[Cu(C_12_H_7_BrClN_2_O)_2_]	*F*(000) = 1340
*M**_r_* = 684.65	*D*_x_ = 1.810 Mg m^−^^3^
Monoclinic, *P*2_1_/*c*	Mo *K*α radiation, λ = 0.71073 Å
Hall symbol: -P 2ybc	Cell parameters from 2128 reflections
*a* = 20.406 (2) Å	θ = 3.5–23.5°
*b* = 11.6378 (15) Å	µ = 4.29 mm^−^^1^
*c* = 10.5787 (13) Å	*T* = 298 K
β = 90.212 (2)°	Needle-like, brown
*V* = 2512.2 (5) Å^3^	0.43 × 0.12 × 0.05 mm
*Z* = 4	

### Data collection

**Table:**

Siemens SMART 1000 CCD area-detector diffractometer	4426 independent reflections
Radiation source: fine-focus sealed tube	2340 reflections with *I* > 2σ(*I*)
graphite	*R*_int_ = 0.060
φ and ω scans	θ_max_ = 25.0°, θ_min_ = 2.0°
Absorption correction: multi-scan (*SADABS*; Sheldrick, 1996)	*h* = −17→24
*T*_min_ = 0.260, *T*_max_ = 0.814	*k* = −13→13
11575 measured reflections	*l* = −11→12

### Refinement

**Table:**

Refinement on *F*^2^	Primary atom site location: structure-invariant direct methods
Least-squares matrix: full	Secondary atom site location: difference Fourier map
*R*[*F*^2^ > 2σ(*F*^2^)] = 0.037	Hydrogen site location: inferred from neighbouring sites
*wR*(*F*^2^) = 0.050	H-atom parameters constrained
*S* = 0.88	*w* = 1/[σ^2^(*F*_o_^2^) + (0.0002*P*)^2^] where *P* = (*F*_o_^2^ + 2*F*_c_^2^)/3
4426 reflections	(Δ/σ)_max_ < 0.001
316 parameters	Δρ_max_ = 0.37 e Å^−^^3^
0 restraints	Δρ_min_ = −0.35 e Å^−^^3^

### Special details

**Table:**

Geometry. All e.s.d.'s (except the e.s.d. in the dihedral angle between two l.s. planes) are estimated using the full covariance matrix. The cell e.s.d.'s are taken into account individually in the estimation of e.s.d.'s in distances, angles and torsion angles; correlations between e.s.d.'s in cell parameters are only used when they are defined by crystal symmetry. An approximate (isotropic) treatment of cell e.s.d.'s is used for estimating e.s.d.'s involving l.s. planes.
Refinement. Refinement of *F*^2^ against ALL reflections. The weighted *R*-factor *wR* and goodness of fit *S* are based on *F*^2^, conventional *R*-factors *R* are based on *F*, with *F* set to zero for negative *F*^2^. The threshold expression of *F*^2^ > σ(*F*^2^) is used only for calculating *R*-factors(gt) *etc*. and is not relevant to the choice of reflections for refinement. *R*-factors based on *F*^2^ are statistically about twice as large as those based on *F*, and *R*- factors based on ALL data will be even larger.

### Fractional atomic coordinates and isotropic or equivalent isotropic displacement parameters (Å^2^)

**Table:**

	*x*	*y*	*z*	*U*_iso_*/*U*_eq_	
Cu1	0.25248 (3)	0.22385 (4)	0.41801 (5)	0.04108 (16)	
Br1	0.13387 (3)	−0.36645 (4)	0.54348 (6)	0.0734 (2)	
Br2	0.35180 (3)	0.81346 (4)	0.23403 (5)	0.06505 (18)	
Cl1	0.37963 (6)	0.12525 (11)	0.20905 (11)	0.0646 (4)	
Cl2	0.20549 (7)	0.12485 (9)	0.15999 (12)	0.0644 (4)	
N1	0.4802 (2)	0.1582 (3)	0.3542 (4)	0.0521 (11)	
N2	0.31148 (17)	0.0920 (3)	0.4578 (3)	0.0363 (9)	
N3	0.0797 (3)	0.1474 (4)	0.1537 (4)	0.0752 (15)	
N4	0.19682 (17)	0.3327 (3)	0.3208 (3)	0.0358 (9)	
O1	0.17657 (13)	0.1421 (2)	0.4686 (3)	0.0435 (8)	
O2	0.32382 (13)	0.3273 (2)	0.4265 (3)	0.0437 (8)	
C1	0.2900 (2)	−0.0104 (3)	0.4797 (3)	0.0356 (11)	
H1	0.3217	−0.0666	0.4935	0.043*	
C2	0.2227 (2)	−0.0477 (3)	0.4853 (4)	0.0366 (12)	
C3	0.1698 (2)	0.0308 (4)	0.4827 (4)	0.0371 (12)	
C4	0.1058 (2)	−0.0135 (3)	0.4983 (4)	0.0480 (13)	
H4	0.0702	0.0364	0.4982	0.058*	
C5	0.0954 (2)	−0.1300 (4)	0.5138 (4)	0.0551 (14)	
H5	0.0530	−0.1578	0.5230	0.066*	
C6	0.1480 (2)	−0.2060 (3)	0.5158 (4)	0.0486 (13)	
C7	0.2108 (2)	−0.1670 (3)	0.5022 (4)	0.0444 (13)	
H7	0.2457	−0.2185	0.5041	0.053*	
C8	0.4174 (2)	0.1324 (3)	0.3550 (4)	0.0378 (12)	
C9	0.3807 (2)	0.1104 (3)	0.4641 (4)	0.0354 (12)	
C10	0.4137 (2)	0.1121 (3)	0.5773 (4)	0.0438 (12)	
H10	0.3916	0.0960	0.6520	0.053*	
C11	0.4796 (2)	0.1377 (4)	0.5798 (5)	0.0547 (14)	
H11	0.5028	0.1389	0.6556	0.066*	
C12	0.5103 (2)	0.1615 (4)	0.4666 (6)	0.0524 (14)	
H12	0.5545	0.1809	0.4688	0.063*	
C13	0.2121 (2)	0.4391 (4)	0.3010 (4)	0.0396 (12)	
H13	0.1802	0.4851	0.2636	0.048*	
C14	0.2736 (2)	0.4933 (3)	0.3312 (4)	0.0353 (11)	
C15	0.3267 (2)	0.4341 (4)	0.3864 (4)	0.0348 (11)	
C16	0.3869 (2)	0.4935 (3)	0.3971 (4)	0.0442 (12)	
H16	0.4226	0.4569	0.4346	0.053*	
C17	0.3941 (2)	0.6050 (4)	0.3531 (4)	0.0505 (14)	
H17	0.4345	0.6416	0.3590	0.061*	
C18	0.3414 (3)	0.6615 (3)	0.3007 (4)	0.0443 (13)	
C19	0.2818 (2)	0.6099 (3)	0.2905 (4)	0.0434 (13)	
H19	0.2464	0.6503	0.2572	0.052*	
C20	0.1335 (2)	0.1942 (4)	0.2005 (5)	0.0523 (14)	
C21	0.1350 (2)	0.2933 (4)	0.2745 (4)	0.0417 (12)	
C22	0.0771 (3)	0.3453 (4)	0.3016 (5)	0.0582 (15)	
H22	0.0757	0.4103	0.3526	0.070*	
C23	0.0194 (3)	0.2987 (5)	0.2509 (6)	0.0788 (18)	
H23	−0.0209	0.3340	0.2644	0.095*	
C24	0.0237 (3)	0.2009 (6)	0.1816 (6)	0.093 (2)	
H24	−0.0151	0.1688	0.1514	0.112*	

### Atomic displacement parameters (Å^2^)

**Table:**

	*U*^11^	*U*^22^	*U*^33^	*U*^12^	*U*^13^	*U*^23^
Cu1	0.0426 (4)	0.0358 (3)	0.0447 (4)	−0.0020 (3)	−0.0014 (3)	0.0047 (3)
Br1	0.0707 (4)	0.0367 (3)	0.1127 (5)	−0.0049 (3)	0.0121 (4)	0.0083 (3)
Br2	0.0813 (5)	0.0403 (3)	0.0736 (4)	−0.0137 (3)	0.0085 (3)	0.0061 (3)
Cl1	0.0733 (10)	0.0825 (9)	0.0381 (8)	−0.0144 (8)	0.0027 (7)	0.0040 (7)
Cl2	0.0823 (11)	0.0519 (7)	0.0589 (9)	−0.0004 (7)	−0.0031 (7)	−0.0107 (7)
N1	0.039 (3)	0.066 (3)	0.051 (3)	−0.008 (2)	0.009 (2)	0.000 (2)
N2	0.041 (3)	0.036 (2)	0.033 (2)	−0.0018 (19)	0.0009 (19)	0.0046 (18)
N3	0.072 (4)	0.074 (3)	0.080 (4)	−0.023 (3)	−0.024 (3)	0.008 (3)
N4	0.041 (3)	0.035 (2)	0.032 (2)	−0.0039 (19)	−0.0021 (19)	0.0037 (18)
O1	0.039 (2)	0.0334 (16)	0.058 (2)	−0.0003 (15)	0.0065 (15)	0.0095 (16)
O2	0.042 (2)	0.0348 (16)	0.054 (2)	−0.0035 (15)	−0.0072 (15)	0.0078 (16)
C1	0.043 (3)	0.036 (3)	0.028 (3)	0.010 (2)	−0.005 (2)	0.001 (2)
C2	0.034 (3)	0.036 (3)	0.039 (3)	−0.003 (2)	0.002 (2)	0.004 (2)
C3	0.042 (3)	0.037 (3)	0.033 (3)	−0.002 (3)	0.005 (2)	0.001 (2)
C4	0.041 (4)	0.038 (3)	0.065 (4)	0.001 (2)	0.003 (3)	0.006 (3)
C5	0.035 (3)	0.048 (3)	0.082 (4)	−0.008 (3)	0.001 (3)	0.003 (3)
C6	0.053 (4)	0.032 (3)	0.061 (4)	−0.005 (3)	0.004 (3)	0.003 (3)
C7	0.050 (4)	0.035 (3)	0.048 (3)	0.006 (2)	0.002 (3)	0.001 (2)
C8	0.045 (3)	0.036 (2)	0.033 (3)	0.006 (2)	0.002 (3)	0.004 (2)
C9	0.035 (3)	0.034 (3)	0.037 (3)	0.001 (2)	−0.001 (3)	−0.005 (2)
C10	0.047 (4)	0.052 (3)	0.032 (3)	0.001 (3)	0.000 (3)	0.008 (3)
C11	0.046 (4)	0.062 (3)	0.055 (4)	0.005 (3)	−0.012 (3)	−0.003 (3)
C12	0.029 (3)	0.051 (3)	0.078 (4)	0.002 (2)	0.009 (3)	−0.006 (3)
C13	0.042 (3)	0.046 (3)	0.031 (3)	0.008 (3)	0.001 (2)	0.008 (2)
C14	0.038 (3)	0.040 (3)	0.027 (3)	0.000 (3)	0.005 (2)	0.001 (2)
C15	0.032 (3)	0.043 (3)	0.029 (3)	−0.006 (3)	0.005 (2)	−0.001 (2)
C16	0.048 (4)	0.045 (3)	0.040 (3)	−0.006 (3)	−0.002 (2)	−0.003 (2)
C17	0.048 (4)	0.051 (3)	0.053 (4)	−0.018 (3)	0.010 (3)	−0.015 (3)
C18	0.053 (4)	0.032 (3)	0.048 (3)	−0.010 (3)	0.007 (3)	0.003 (2)
C19	0.054 (4)	0.032 (3)	0.044 (3)	0.006 (2)	0.006 (3)	0.002 (2)
C20	0.054 (4)	0.052 (3)	0.051 (4)	−0.017 (3)	−0.012 (3)	0.015 (3)
C21	0.042 (4)	0.045 (3)	0.038 (3)	−0.010 (3)	−0.007 (3)	0.008 (3)
C22	0.043 (4)	0.062 (3)	0.070 (4)	−0.001 (3)	−0.004 (3)	0.009 (3)
C23	0.048 (4)	0.094 (5)	0.094 (5)	0.001 (4)	−0.003 (4)	0.033 (4)
C24	0.062 (5)	0.106 (6)	0.110 (6)	−0.044 (5)	−0.039 (4)	0.026 (5)

### Geometric parameters (Å, °)

**Table:**

Cu1—O2	1.891 (3)	C6—C7	1.368 (5)
Cu1—O1	1.897 (2)	C7—H7	0.9300
Cu1—N4	1.986 (3)	C8—C9	1.402 (5)
Cu1—N2	1.994 (3)	C9—C10	1.372 (5)
Br1—C6	1.912 (4)	C10—C11	1.378 (5)
Br2—C18	1.916 (4)	C10—H10	0.9300
Cl1—C8	1.726 (4)	C11—C12	1.381 (5)
Cl2—C20	1.731 (5)	C11—H11	0.9300
N1—C8	1.316 (5)	C12—H12	0.9300
N1—C12	1.336 (6)	C13—C14	1.439 (5)
N2—C1	1.291 (4)	C13—H13	0.9300
N2—C9	1.429 (5)	C14—C15	1.410 (5)
N3—C20	1.320 (5)	C14—C19	1.434 (5)
N3—C24	1.335 (6)	C15—C16	1.415 (5)
N4—C13	1.295 (4)	C16—C17	1.387 (5)
N4—C21	1.427 (5)	C16—H16	0.9300
O1—C3	1.311 (4)	C17—C18	1.376 (6)
O2—C15	1.314 (4)	C17—H17	0.9300
C1—C2	1.442 (5)	C18—C19	1.361 (5)
C1—H1	0.9300	C19—H19	0.9300
C2—C3	1.414 (5)	C20—C21	1.395 (6)
C2—C7	1.421 (5)	C21—C22	1.359 (5)
C3—C4	1.416 (5)	C22—C23	1.400 (6)
C4—C5	1.382 (5)	C22—H22	0.9300
C4—H4	0.9300	C23—C24	1.358 (7)
C5—C6	1.390 (5)	C23—H23	0.9300
C5—H5	0.9300	C24—H24	0.9300
			
O2—Cu1—O1	159.31 (12)	C9—C10—H10	120.2
O2—Cu1—N4	93.27 (13)	C11—C10—H10	120.2
O1—Cu1—N4	89.99 (13)	C10—C11—C12	118.2 (5)
O2—Cu1—N2	90.91 (13)	C10—C11—H11	120.9
O1—Cu1—N2	92.73 (13)	C12—C11—H11	120.9
N4—Cu1—N2	160.68 (13)	N1—C12—C11	123.8 (5)
C8—N1—C12	116.4 (4)	N1—C12—H12	118.1
C1—N2—C9	117.8 (3)	C11—C12—H12	118.1
C1—N2—Cu1	122.9 (3)	N4—C13—C14	126.4 (4)
C9—N2—Cu1	119.3 (2)	N4—C13—H13	116.8
C20—N3—C24	115.8 (5)	C14—C13—H13	116.8
C13—N4—C21	117.7 (4)	C15—C14—C19	119.8 (4)
C13—N4—Cu1	123.8 (3)	C15—C14—C13	123.1 (4)
C21—N4—Cu1	118.4 (3)	C19—C14—C13	116.8 (4)
C3—O1—Cu1	127.9 (3)	O2—C15—C14	124.1 (4)
C15—O2—Cu1	128.5 (3)	O2—C15—C16	118.4 (4)
N2—C1—C2	127.5 (4)	C14—C15—C16	117.4 (4)
N2—C1—H1	116.2	C17—C16—C15	121.6 (4)
C2—C1—H1	116.2	C17—C16—H16	119.2
C3—C2—C7	120.2 (4)	C15—C16—H16	119.2
C3—C2—C1	122.1 (4)	C18—C17—C16	119.9 (4)
C7—C2—C1	117.5 (4)	C18—C17—H17	120.0
O1—C3—C2	124.0 (4)	C16—C17—H17	120.0
O1—C3—C4	118.1 (4)	C19—C18—C17	121.3 (4)
C2—C3—C4	117.9 (4)	C19—C18—Br2	118.6 (4)
C5—C4—C3	120.8 (4)	C17—C18—Br2	120.1 (4)
C5—C4—H4	119.6	C18—C19—C14	119.9 (4)
C3—C4—H4	119.6	C18—C19—H19	120.0
C4—C5—C6	120.5 (4)	C14—C19—H19	120.0
C4—C5—H5	119.7	N3—C20—C21	124.6 (5)
C6—C5—H5	119.7	N3—C20—Cl2	114.8 (5)
C7—C6—C5	120.7 (4)	C21—C20—Cl2	120.5 (4)
C7—C6—Br1	118.8 (3)	C22—C21—C20	118.0 (4)
C5—C6—Br1	120.4 (4)	C22—C21—N4	123.6 (4)
C6—C7—C2	119.8 (4)	C20—C21—N4	118.4 (4)
C6—C7—H7	120.1	C21—C22—C23	118.5 (5)
C2—C7—H7	120.1	C21—C22—H22	120.7
N1—C8—C9	124.8 (4)	C23—C22—H22	120.7
N1—C8—Cl1	115.9 (3)	C24—C23—C22	118.4 (6)
C9—C8—Cl1	119.3 (4)	C24—C23—H23	120.8
C10—C9—C8	117.0 (4)	C22—C23—H23	120.8
C10—C9—N2	121.6 (4)	N3—C24—C23	124.6 (6)
C8—C9—N2	121.4 (4)	N3—C24—H24	117.7
C9—C10—C11	119.7 (4)	C23—C24—H24	117.7
			
O2—Cu1—N2—C1	172.5 (3)	C1—N2—C9—C10	−72.8 (5)
O1—Cu1—N2—C1	12.8 (3)	Cu1—N2—C9—C10	106.5 (4)
N4—Cu1—N2—C1	−84.9 (5)	C1—N2—C9—C8	109.8 (4)
O2—Cu1—N2—C9	−6.8 (3)	Cu1—N2—C9—C8	−70.8 (4)
O1—Cu1—N2—C9	−166.4 (3)	C8—C9—C10—C11	1.7 (6)
N4—Cu1—N2—C9	95.8 (5)	N2—C9—C10—C11	−175.7 (4)
O2—Cu1—N4—C13	−9.1 (3)	C9—C10—C11—C12	0.2 (6)
O1—Cu1—N4—C13	150.5 (3)	C8—N1—C12—C11	1.1 (7)
N2—Cu1—N4—C13	−111.3 (5)	C10—C11—C12—N1	−1.8 (7)
O2—Cu1—N4—C21	173.9 (3)	C21—N4—C13—C14	−175.0 (4)
O1—Cu1—N4—C21	−26.6 (3)	Cu1—N4—C13—C14	7.9 (6)
N2—Cu1—N4—C21	71.7 (5)	N4—C13—C14—C15	0.9 (7)
O2—Cu1—O1—C3	−117.9 (4)	N4—C13—C14—C19	174.6 (4)
N4—Cu1—O1—C3	142.8 (4)	Cu1—O2—C15—C14	2.2 (6)
N2—Cu1—O1—C3	−18.0 (4)	Cu1—O2—C15—C16	−176.8 (3)
O1—Cu1—O2—C15	−94.3 (5)	C19—C14—C15—O2	180.0 (3)
N4—Cu1—O2—C15	4.4 (3)	C13—C14—C15—O2	−6.6 (6)
N2—Cu1—O2—C15	165.5 (3)	C19—C14—C15—C16	−1.0 (6)
C9—N2—C1—C2	176.4 (4)	C13—C14—C15—C16	172.4 (4)
Cu1—N2—C1—C2	−2.9 (6)	O2—C15—C16—C17	177.9 (4)
N2—C1—C2—C3	−8.4 (7)	C14—C15—C16—C17	−1.2 (6)
N2—C1—C2—C7	175.7 (4)	C15—C16—C17—C18	1.8 (6)
Cu1—O1—C3—C2	12.6 (6)	C16—C17—C18—C19	−0.2 (7)
Cu1—O1—C3—C4	−168.5 (3)	C16—C17—C18—Br2	−177.7 (3)
C7—C2—C3—O1	179.4 (4)	C17—C18—C19—C14	−2.0 (7)
C1—C2—C3—O1	3.6 (7)	Br2—C18—C19—C14	175.5 (3)
C7—C2—C3—C4	0.5 (6)	C15—C14—C19—C18	2.6 (6)
C1—C2—C3—C4	−175.3 (4)	C13—C14—C19—C18	−171.3 (4)
O1—C3—C4—C5	−179.9 (4)	C24—N3—C20—C21	−0.3 (7)
C2—C3—C4—C5	−0.9 (6)	C24—N3—C20—Cl2	−178.6 (4)
C3—C4—C5—C6	0.8 (7)	N3—C20—C21—C22	0.6 (7)
C4—C5—C6—C7	−0.2 (7)	Cl2—C20—C21—C22	178.8 (3)
C4—C5—C6—Br1	177.7 (3)	N3—C20—C21—N4	178.7 (4)
C5—C6—C7—C2	−0.3 (7)	Cl2—C20—C21—N4	−3.0 (5)
Br1—C6—C7—C2	−178.1 (3)	C13—N4—C21—C22	−53.4 (6)
C3—C2—C7—C6	0.1 (7)	Cu1—N4—C21—C22	123.8 (4)
C1—C2—C7—C6	176.1 (4)	C13—N4—C21—C20	128.5 (4)
C12—N1—C8—C9	1.1 (6)	Cu1—N4—C21—C20	−54.3 (5)
C12—N1—C8—Cl1	−178.6 (3)	C20—C21—C22—C23	−1.8 (7)
N1—C8—C9—C10	−2.5 (6)	N4—C21—C22—C23	−179.9 (4)
Cl1—C8—C9—C10	177.1 (3)	C21—C22—C23—C24	2.8 (8)
N1—C8—C9—N2	174.9 (4)	C20—N3—C24—C23	1.4 (9)
Cl1—C8—C9—N2	−5.4 (5)	C22—C23—C24—N3	−2.7 (9)
